# Scaling evidence-based interventions: Examining factors promoting and limiting the dissemination of research mentor training

**DOI:** 10.1017/cts.2025.41

**Published:** 2025-02-26

**Authors:** Kimberly Spencer, Melissa McDaniels, So Hee Hyun, Jenna Griebel Rogers, Emily Utzerath, Christine Pfund

**Affiliations:** 1 Center for the Improvement of Mentored Experiences in Research, Wisconsin Center for Education Research, University of Wisconsin–Madison, Madison, WI, USA; 2 Institute for Clinical and Translational Research, University of Wisconsin–Madison, Madison, WI, USA

**Keywords:** Mentor training, capacity-building, implementation, facilitator training, dissemination

## Abstract

**Introduction:**

Mentorship education has been shown to positively impact the experiences of mentors and mentees. *Entering Mentoring*, an evidence-based mentor training curriculum, has been widely implemented to train research mentors across the country, including the mentors of clinical and translational scientists. *Facilitating Entering Mentoring*, a train-the-trainer based workshop, has been used as a dissemination strategy to increase the number of facilitators prepared to implement mentor training in their local contexts. The objective of this research was to examine individual and institutional factors promoting and limiting mentor training implementation efforts of trained facilitators.

**Methods:**

Using the Consolidated Framework for Implementation Research (CFIR), we examined self-reported data from surveys administered annually to *Facilitating Entering Mentoring* participants. Data analyses included *t*-tests to compare differences between the implementer and non-implementer groups and binary logistic regression to determine which factors best predict implementation status.

**Results:**

Factors associated with the inner setting domain were found to have the most impact on implementation efforts, with administrative support, leadership support, and interest from potential participants being the most significant predictors of implementation. Additionally, those who implemented were more likely to report receiving institutional support compared with those who did not implement the intervention. Those who did not implement were more likely to report the presence of perceived institutional barriers.

**Conclusions:**

The CFIR model provides a useful framework for understanding factors that promote and limit implementation outcomes of an evidence-based research mentor training intervention. Findings emphasize the role of institutional support to promote the implementation of research mentor training.

## Introduction

The importance of strong mentorship has been shown to have benefits for both the mentor and mentee [1]. Effective mentoring is also an important predictor of trainee persistence and degree attainment [2], including those in STEM and health science programs [3]. The evidence-based research mentor training intervention, *Entering Mentoring*, is one approach to formal mentorship education [4,5].

*Entering Mentoring* is designed for research mentors across career stages [6–10], including the mentors of clinical and translational researchers, and has been shown to have a significant positive impact on mentor self-perceived skills, the mentee’s perception of their mentor’s skills, and on the overall research mentoring relationship [11–15].

This manuscript builds upon previously published work [11–13] to further examine the impact of a train-the-trainer workshop, *Facilitating Entering Mentoring*, as a strategy to increase the number of facilitators who are prepared to lead *Entering Mentoring* mentor training workshops in their local contexts.

### Facilitating Entering Mentoring for national dissemination

To disseminate *Entering Mentoring* on a national scale, a train-the-trainer workshop, *Facilitating Entering Mentoring*, was developed [11–13]. Train-the-trainer workshops are a common mechanism for scaling-up evidence-based interventions that has proven to be highly effective at increasing participant knowledge, skills, and confidence to implement interventions in educational contexts [16,17]. This model was designed to increase the number of trained facilitators prepared to lead mentor training for faculty, postdocs, and graduate students, thus amplifying the number of research mentors receiving formal mentorship education and broadening access to high-quality mentorship. The *Facilitating Entering Mentoring* workshop has been studied over time and is effective across multiple audiences, with participants reporting high satisfaction and significant confidence gains [12,13].

### Our dissemination model situated in the implementation science literature

**Figure 1.**
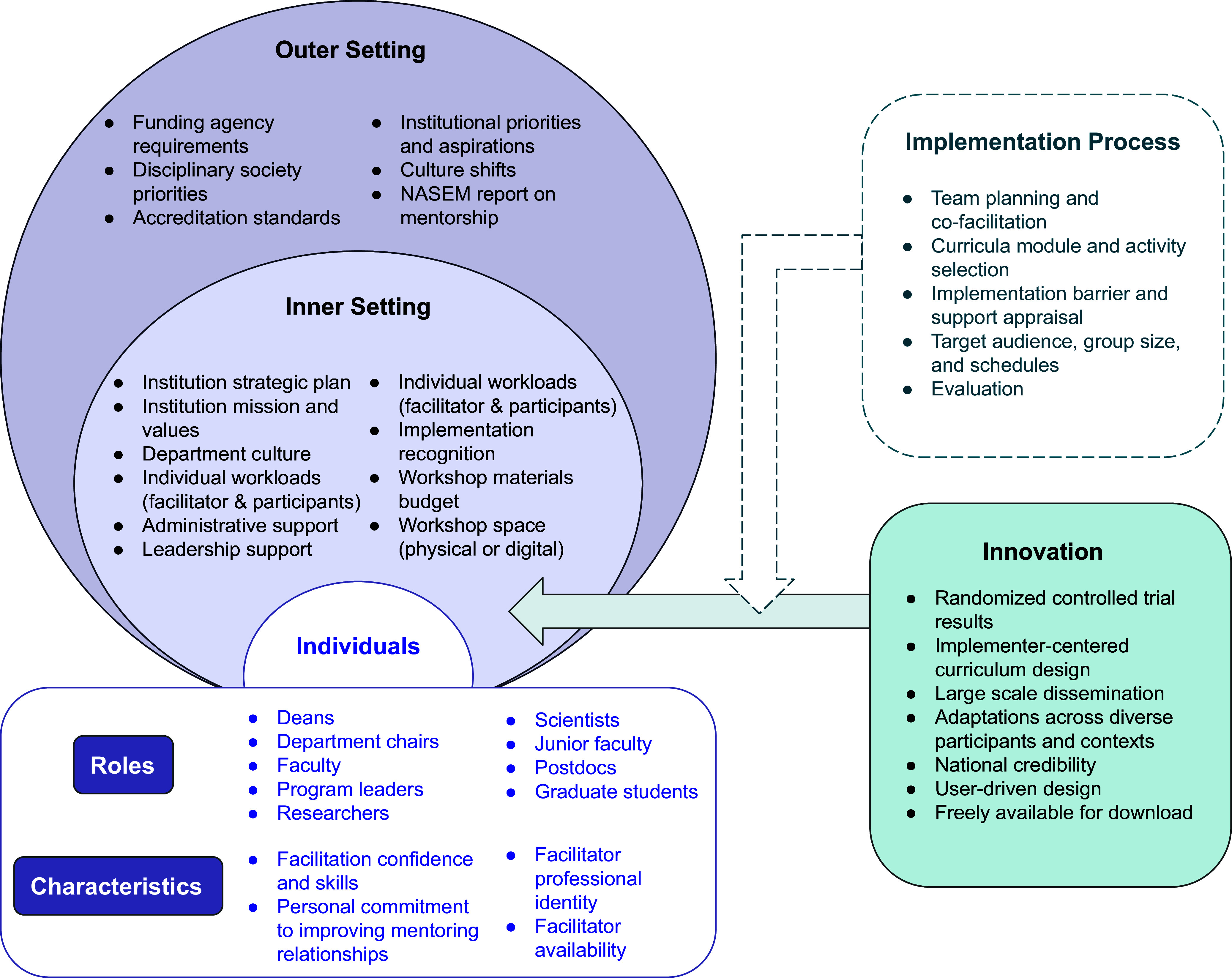
Consolidated Framework for Implementation Research (CFIR) 2.0 for *Entering Mentoring* ^[48].^ NASEM = National Academies of Sciences, Engineering, and Medicine.

The field of dissemination and implementation research offers valuable literature and frameworks for understanding the challenges and opportunities in our efforts to disseminate the *Entering Mentoring* intervention. Applied retroactively, the Consolidated Framework for Implementation Research (CFIR) can be used to highlight the constructs most relevant to our dissemination efforts before examining implementation outcomes [18,19]. The five core CFIR domains are outlined in Table 1 with select constructs integrated in Figure 1.

**Table 1.** Consolidated Framework for Implementation Research (CFIR) domains and constructs for the *Entering Mentoring* innovation

Domain	Definition
Innovation	The “thing” being implemented
Individuals	The roles and characteristics of individuals involved with implementing, delivering, and/or receiving the innovation
Implementation process	The activities and strategies used to implement the innovation
Inner setting	The setting in which the innovation is implemented (e.g., hospital, school, city). There may be multiple inner settings and/or multiple levels within the inner setting (e.g., unit, classroom, team)
Outer setting	The setting in which the inner setting exists (e.g., hospital system, school district, state). There may be multiple outer settings and/or multiple levels within the outer setting (e.g., community, system, state)

**Domain: Innovation.** The innovation [18,20] at the heart of this work is *Entering Mentoring*, a research mentor training curriculum, which has been studied consistently over the past 15 years. The *trialability* and *evidence-base* for the effectiveness and efficacy [21] of *Entering Mentoring* is robust and has been demonstrated through a double-blind randomized controlled trial [11]. The original developers of the *Entering Mentoring* curriculum are national experts in mentorship education, contributing to the *innovation source*. Since 2005, *Entering Mentoring* curricula have been used to train research mentors across the country. Additionally, important stakeholders, including the Howard Hughes Medical Institute, the National Institutes of Health (NIH), and the National Academies of Sciences, Engineering, and Medicine (NASEM) have noted the power of this approach and have highlighted it in a number of publications [1,22–25].

*Entering Mentoring* is also noted for its *adaptability* with over 15 versions created for use with mentors across multiple career stages and disciplines, including *Mentor Training for Clinical and Translational Researchers*, which is implemented widely across institutions with Clinical and Translational Science Awards [6,26]. Contributing to the innovation *design*, the curriculum is easily segmented to align with learning goals and includes detailed facilitation notes to encourage widespread use. The curriculum itself is freely accessible (*innovation cost*) through the Center for the Improvement of Mentored Experiences in Research (CIMER) [27] online portal.

**Domain: Individuals.** Many individuals are ultimately responsible (directly or indirectly) for successful innovation implementation [18]. Individuals who become trained facilitators through attending a *Facilitating Entering Mentoring* workshop return to their home institutions as *innovation deliverers* with the skills, access to materials and networks, commitment and knowledge, and beliefs about the innovation to launch a successful implementation.

The innovation deliverers will not succeed in a vacuum. *High- and mid-level leaders* at colleges and universities (including provosts, deans, and department chairs) may make critical decisions about funding for implementation and play an important role in communicating the value of this training for the entire community. Institutional leaders and advocates willing to invest in the implementation of *Entering Mentoring* workshops are key, and data in our paper will support this. Finally, successful implementation will only happen with mentors, the *innovation recipients*, who are willing to participate in these innovations.

The professional roles, identities, skills, and level of involvement of innovation deliverers are also important aspects of the implementation process. These facilitators will assess their confidence and skills as *implementation leaders* (*capability*), their own availability and the availability of *innovation recipients* (*opportunity*), as well as the connection to feeling fulfilled and having a personal commitment to improving mentoring relationships (*need, motivation*).

**Domain: Implementation Process**. The implementation process for *Entering Mentoring* relies heavily upon the individuals who decide to implement the innovation and the activities and strategies used to implement the innovation [18]. The *Facilitating Entering Mentoring* workshop is utilized to support the implementation of *Entering Mentoring* [12,13]. Importantly, the workshop content and learning objectives of *Facilitating Entering Mentoring* remain the same and are applicable for implementation of all *Entering Mentoring* adaptations and delivery modalities (*assessing context*).

Individuals are strongly encouraged to attend *Facilitating Entering Mentoring* with colleagues from their own institution, professional community, or disciplinary society. This *teaming* allows for participants to jointly experience the curriculum, *assess the needs* of innovation recipients, and develop an implementation *plan* to confirm roles and responsibilities, address anticipated barriers and supports, and identify strategies for participant engagement.

*Facilitating Entering Mentoring* workshop participants receive access to many implementation resources, including curricular adaptations, facilitation experts, evaluation tools, and membership in a national community of facilitators with whom to share ideas and best practices. These resources are designed to support the modification of the innovation for different audiences (*adapting*), optimize delivery of the innovation (*doing*), and evaluate the implementation (*reflecting and evaluating*).

**Domain: Inner Setting.** The inner setting where *Entering Mentoring* is implemented is the organizational unit(s) within colleges and universities (departments, centers, administrative units, and comparable units outside the academy). Within the organizational unit, team member tasks, responsibilities, incentives (*work infrastructure*), and shared beliefs and norms (*culture*) influence implementation outcomes. The degree to which an organization explicitly values and invests in its human resources will impact, through *incentive and other reward systems*, whether implementation occurs. Many organizations, through observation about graduate student and faculty attrition, are realizing that the “time is now” to directly address mentorship needs. This tension for change, combined with an organization’s explicit *mission-driven commitments* to student success and research quality, can drive support for department and institution-wide mentorship programs and initiatives.

The resources provided by the institution, including financial funding, physical space, and materials (*structural characteristics*), also contribute to implementation. To address differences in inner setting variation, resources on strategies for securing stakeholder support are integrated into the *Facilitating Entering Mentoring* workshop. However, as the data in this paper shows, the inner setting is complex and has a great influence on implementation outcomes.

**Domain: Outer Setting.** Outer setting factors external to the organization or unit where the implementation is being attempted will impact the success of an implementation effort [18]. On the national level, *critical incidents* including a public health crisis (e.g., COVID-19), racially motivated hate crimes, policy changes, and other large-scale incidents will disrupt or limit the implementation of *Entering Mentoring* workshops. Likewise, *external partnerships and funders* can have a significant leverage in supporting or detracting from implementation efforts.

In recent years, funding agencies such as the NIH [28] the National Science Foundation [29] (*local and federal policies and laws*) and private foundations such as the Howard Hughes Medical Institute [24] and the Alfred P. Sloan Foundation [30] have begun recommending or even requiring formalized mentorship education for faculty of trainees in training grants and research programs. Similarly, in recent years, the National Academies of Sciences, Engineering, and Medicine (NASEM) has convened researchers and practitioners across the country to curate what has been learned and what is not yet known about advancing equity in STEM generally and more specifically the important role mentorship plays in these efforts. Two reports, *The Science of Mentorship in STEMM* [1] and *Advancing Antiracism, Diversity, Equity and Inclusion in STEMM Organizations* [31], a national leadership summit [32], as well as a roundtable [33] have amplified the work that still needs to be addressed. These efforts fully recognized the importance of broadening access to high-quality mentorship. Increasing calls for mentorship education have also been more prevalent, noting the importance of mentoring for trainee persistence and career advancement [22,34–36]. These federal agencies and organizations contribute to institutional *external pressure* (or lack thereof) from peers to influence the implementation of mentor training innovations.

### Factors promoting and limiting the dissemination of research mentor training

This paper examines variables among *Facilitating Entering Mentoring* participants who reported locally implementing research mentor training, hereafter referred to as the “implementer group” compared with those who reported no implementation efforts, referred to as the “non-implementer group.” These data are examined in the context of CFIR to explore factors that promote and limit implementation outcomes and advance the culture of mentorship.

The empirical questions explored in this paper include:

- What individual factors impact the implementation of research mentor training?
- What institutional support and barrier factors associated with the inner setting did facilitators experience during the implementation planning process?
- How did these factors vary between the implementer and non-implementer groups?

## Method

### Consolidated Framework for Implementation Research

The CFIR is used retrospectively to explore national dissemination efforts, situate our survey design, and frame the results of our study.

### Intervention implementation survey

An Intervention Implementation Survey was administered annually from Spring 2016 to Spring 2020 to individuals who attended a *Facilitating Entering Mentoring* workshop. The Intervention Implementation Survey was used to collect data on (1) whether participants implemented mentor training since attending the *Facilitating Entering Mentoring* workshop, (2) factors impacting their initial implementation of mentor training since attending the workshop, (3) the impact of perceived institutional supports on their implementation efforts, and (4) the impact of perceived institutional barriers on their implementation efforts. Demographic questions in the survey were chosen to align with the questions and categories used by the NIH and in the US census survey at the time the survey was created. During analysis, academic titles were recategorized to reflect standard title structures across academic institutions.

### Quantitative Analyses

An independent samples *t*-test was used to compare the means of the implementer and non-implementer group to determine whether the associated population means are significantly different across individual factors, institutional support factors, and institutional barrier factors. Based on the *t*-test results, a binary logistic regression was performed to determine which factors best predicted implementation status. A binary logistic regression was determined most appropriate since the dependent variable (implementer vs non-implementer group) was binary. Demographic variables (race/ethnicity, title, and gender), individual factors, institutional supports, and institutional barriers were included in the model as independent variables.

## Results

An Intervention Implementation Survey was administered from Spring 2016 to Spring 2020 to 1346 *Facilitating Entering Mentoring* workshop participants, and responses were collected from 405 individuals across 145 institutions, including 54 institutions associated with Clinical and Translational Science hubs, for a response rate of 30.09%. The characteristics of respondents, including gender identity, race, ethnicity, and title, are described in Table 2. The majority of respondents identified as female (55.4%), White (62.0%), and non-Hispanic or Latino (92.4%). This demographic makeup of the respondents mirrors the demographic distribution of the *Facilitating Entering Mentoring* attendees overall and is similar to the demographics of non-respondents. Survey questions were optional, and while all survey respondents indicated their title, a group of respondents did not provide gender or race/ethnicity demographic information. Across all survey respondents, 72.6% reported implementing mentor training since attending the *Facilitating Entering Mentoring* workshop (implementer group), and 27.5% reported no implementation efforts since the workshop (non-implementer group).

**Table 2.** Intervention implementation survey respondent demographic characteristics

Demographic Characteristics		
**Gender identity ^a^ (choose all that apply)**	**Frequency**	**Percent**
Male	104	25.5
Female	226	55.4
Prefer not to report	4	1.0
**Race (choose all that apply)**	**Frequency**	**Percent**
American Indian or Alaskan Native	7	1.7
Asian	26	6.4
Black or African American	40	9.8
Native Hawaiian or Pacific Islander	2	0.5
White	253	62.0
Hispanic or Latino	31	7.6
Other (please specify)	13	3.2
Prefer not to report	5	1.2
**Title^b^ (choose all that apply)**	**Frequency**	**Percent**
Professor	259	63.5
Scientist or researcher	20	4.9
Academic leader	53	13.0
Instructor	25	6.1
Training program leadership	61	15.0
Academic staff	71	17.4
Graduate student	3	0.7
Postdoctoral fellow	9	2.2

a The following gender identity response options were not selected by participants and therefore not included in the table: transgender, intersex, and other.

b Titles were recategorized into new groupings to reflect standard title structures across academic institutions.

Facilitators who completed the annual Intervention Implementation Survey self-rated the quality of their implementations. The majority of facilitators rated their implementation quality as very high or high, with 41 (10%) reporting very high quality, 158 (38.7%) high quality, 67 (16.4%) average, 2 (0.4%) low or very low. While mentor training outcome data are not the focus of this manuscript, initial analyses from mentor training participants indicate high workshop satisfaction, and many mentors report increases in mentoring skills gains and specific plans to make changes in their mentoring relationship. Although incomplete, these data support the effectiveness of the *Facilitating Entering Mentoring* workshop as a mechanism to disseminate evidence-based mentor training on a national level, which has been previously shown through other research [12,13,26].

### Individual implementation factors, perceived inner setting support and barrier factors, and implementation status

Respondents were asked whether they had implemented mentor training since attending the *Facilitating Entering Mentoring* workshop and to rate the degree to which certain individual and inner institutional factors impacted initial implementation efforts. Responses were analyzed by implementation status: the implementer group who facilitated mentor training and the non-implementer group who had not facilitated mentor training. To better understand the differences between those who implemented the intervention and those who did not, *individual factors* in initial implementation efforts were examined. Survey respondents were asked to rate whether each factor positively or negatively impacted their initial implementation efforts. Between the implementer and non-implementer groups, none of the explored factors were identified as having a significantly positive impact on whether they implemented research mentor training (Table 3).

**Table 3.** Individual implementation factors with a positive impact on mentor training implementation

	Implementer Group *N* = 288	Non-implementer Group *N* = 117
**Individual Implementation Factor**	Mean (SD)	Mean (SD)
Confidence in your ability to facilitate training	0.46 (0.50)	0.43 (0.50)
Interest in facilitating training	0.55 (0.50)	0.50 (0.50)
Individuals to co-facilitate trainings with me	0.41 (0.49)	0.42 (0.50)
Access to materials to facilitate training	0.54 (0.50)	0.55 (0.50)
Potential participants available to take training	0.40 (0.49)	0.38 (0.49)
Impact that facilitating would have on my own career goals	0.36 (0.48)	0.38 (0.49)

Participants were asked whether each factor had a positive or negative impact on their initial implementation. Differences were determined using an independent samples *t*-test between the implementer group and non-implementer group using mean (SD) scores with *p < 0.05, **0.001<p < 0.01, ***p < 0.00.

*Institutional inner setting support factors* were examined between the implementer and non-implementer groups. Survey respondents were asked whether their institution provided each type of support. Between the two groups, the implementer group was more likely to report receiving institutional support, including protected time for implementation (*p* < 0.01), administrative support for both recruitment (*p* < 0.00) and logistics (*p* < .05), publicly communicated support (*p* < 0.01), support with identifying participants (*p* < 0.00), monetary compensation for facilitators (*p* < 0.00), monetary compensation for workshop participants (*p* < 0.01), support from institutional leadership (*p* < 0.00), buy-in from colleagues (*p* < 0.00), and significant interest on the part of potential participants (*p* < .05) (Table 4). The non-implementer group was less likely to report receiving institutional support, although more non-implementers reported that their institution would provide recognition of implementation efforts during promotion processes (*p* < 0.05).

**Table 4.** Perceived institutional inner setting factors promoting implementation of mentor training

	Implementer Group *N* = 288	Non-implementer Group *N* = 117
**Institutional Inner Setting Support**	Mean (SD)	Mean (SD)
Recognition of my implementation efforts during merit-raise processes	0.12 (0.32)	0.12 (0.33)
Recognition of my implementation efforts during promotion processes	0.13 (0.34)	0.20** (0.40)
Recognition of my implementation efforts during annual review processes	0.37 (0.48)	0.38 (0.49)
Protected time (within existing role) for implementation	0.25** (0.44)	0.18 (0.39)
Protected time for participating individuals (within their existing roles)	0.16 (0.37)	0.14 (0.35)
Administrative support (e.g., materials, food, space) for training	0.69* (0.46)	0.37 (0.48)
Administrative support for recruitment	0.47*** (0.50)	0.30 (0.46)
Publicly communicated support for implementation	0.45** (0.50)	0.38 (0.49)
Helped you identify training participants	0.48*** (0.50)	0.34 (0.48)
Significant buy-in from colleagues	0.44*** (0.50)	0.31 (0.46)
Significant interest on the part of potential participants	0.61* (0.49)	0.33 (0.47)
Monetary compensation to facilitators	0.10*** (0.31)	0.04 (0.20)
Support from leadership	0.30*** (0.46)	0.20 (0.40)
Monetary compensation to participating individuals	0.06** (0.24)	0.03 (0.16)
Helped you identify co-facilitators	0.25 (0.44)	0.26 (0.44)
Other support	0.07* (0.26)	0.04 (0.20)

Participants were asked about the ways their institution supported implementation efforts. Differences were determined using an independent samples *t*-test between the implementer group and non-implementer group using mean (SD) scores with *p < 0.05, **0.001<p < 0.01, ***p < 0.00.

The implementer group was also more likely to report receiving “Other support.” Open-ended responses to this item included examples such as support from other departments on campus, external grant funding, campus initiatives promoting mentorship, and involvement from administrative leaders. Lastly, *institutional inner setting barrier factors* were examined to determine whether there were differences between the implementer and non-implementer groups. Survey respondents were asked whether they experienced specific barriers at their institution during the mentor training implementation planning process. The majority of institutional barriers listed in the survey were more likely to be reported by the non-implementer group as factors in their implementation planning (Table 5), with several factors being statistically significant. The non-implementer group was also more likely to report experiencing “Other barriers” in their implementation planning. Open-ended responses to this item include examples such as lack of centralized coordination, misalignment with unit/department mission, and an emphasis on lack of time and being stretched too thin.

**Table 5.** Perceived institutional inner setting factors limiting the implementation of mentor training

	Implementer Group *N* = 288	Non-Implementer Group *N* = 117
**Institutional Inner Setting Barrier**	Mean (SD)	Mean (SD)
Lack of recognition of my implementation efforts during merit-raise processes	0.11 (0.32)	0.15 (0.35)
Lack of recognition of my implementation efforts during promotion processes	0.10 (0.30)	0.14* (0.35)
Lack of recognition of my implementation efforts during annual review processes	0.09 (0.29)	0.14** (0.35)
Lack of protected time (within my existing role) for implementation	0.26 (0.44)	0.36*** (0.48)
Lack of protected time (within their existing roles) for participating individuals	0.27 (0.45)	0.35** (0.48)
Lack of administrative support for training (e.g., materials, food, space)	0.08 (0.28)	0.23*** (0.42)
Lack of administrative support for recruitment	0.10 (0.30)	0.21*** (0.41)
No publicly communicated support for implementation	0.12 (0.33)	0.18** (0.39)
No assistance identifying training participants	0.09 (0.29)	0.15** (0.36)
Lack of buy-in from colleagues	0.18 (0.39)	0.22 (0.42)
Lack of interest on the part of potential participants	0.19 (0.40)	0.23 (0.42)
Lack of monetary compensation to facilitators	0.13 (0.34)	0.23*** (0.42)
Lack of support from leadership	0.14 (0.35)	0.17 (0.38)
Other competing trainings offered on campus	0.13 (0.34)	0.18* (0.39)
Lack of monetary compensation to participating individuals	0.11 (0.32)	0.18*** (0.39)
No assistance identifying co-facilitators	0.04 (0.20)	0.09*** (0.29)
Other barriers	0.02 (0.15)	0.09*** (0.29)

Participants were asked whether they experienced barriers at their institution during the mentor training implementation planning process. Differences were determined using an independent samples *t*-test between the implementer group and non-implementer group using mean (SD) scores with *p < 0.05, **0.001<p < 0.01, ***p < 0.00.

### Demographic, individual, and inner setting factors predicting implementation

Binary regression modeling was used to further determine which factors best predicted implementation status. Overall, the entire model was significant *X* ^2^ = 105.037, *p* < .000. Cox & Snell was 0.228, and Negelkerke was 0.327, indicating that the model can discriminate between the implementer and non-implementor group. The model correctly classified 78.8% of the cases.

*Demographic and background factors* used in the model include historically and systemically excluded groups (Black or African American, Native Hawaiian or Pacific Islander, Hispanic or Latino) title, training stage, and gender, which were analyzed to explore whether certain groups were more or less likely to implement (Table 6). Across these variables, two groups were significantly less likely to implement: instructors and postdoctoral fellows. Individual implementation survey items were examined in the model, and none of these factors were found significant (Table 6). These findings are consistent with the results shown in Table 3.

**Table 6.** Binary regression results of demographic, individual, and inner setting variables

Predictor	B (SE)	Wald	Exp(B)
**Demographic/Background Variable**			
Race: Historically excluded groups	−0.254 (0.372)	0.467	0.776
Title: Professor	−0.550 (0.440)	1.562	0.577
Title: Scientist researcher	1.391 (0.883)	2.482	4.018
Title: Academic leader	0.494 (0.436)	1.283	0.610
Title: Instructor	−1.616 (0.651)*	6.163	0.199
Title: Training program leadership	0.394 (0.443)	0.788	0.675
Title: Academic staff	−0.495 (0.488)	1.027	0.610
Training stage: Graduate student	−0.413 (1.480)	0.078	1.512
Training stage: Postdoctoral fellow	−2.166 (1.035)*	4.384	0.115
Gender: Female	0.159 (0.297)	0.284	1.172
**Individual Implementation Factor Variable**			
Confidence in your ability to facilitate training	0.542 (0.391)	1.927	1.720
Interest in facilitating training	0.692 (0.491)	1.986	1.997
Individuals to co-facilitate trainings with me	−0.449 (0.410)	1.197	0.638
Access to materials to facilitate training	−1.003 (0.584)	2.950	0.367
Potential participants available to take training	−0.036 (0.373)	0.009	0.964
Impact that facilitating would have on my own career goals	−0.370 (0.371)	0.993	0.691
**Institutional Inner Setting Support Variable**			
Recognition of my implementation efforts during merit-raise processes	0.495 (0.504)	0.965	1.640
Recognition of my implementation efforts during promotion processes	−0.624 (0.451)	1.919	0.536
Recognition of my implementation efforts during annual review processes	−0.743 (0.380)	3.820	0.476
Protected time (within existing role) for implementation	−0.121 (0.438)	0.076	0.886
Protected time for participating individuals (within their existing roles)	−0.105 (0.462)	0.052	0.900
Administrative support for training (e.g., materials, food, space)	1.408 (0.422)**	11.110	4.089
Administrative support for recruitment	0.121 (0.394)	0.094	1.128
Publicly communicated support for implementation	−0.607 (0.375)	2.620	0.545
Helped you identify training participants	0.458 (0.368)	1.547	1.581
Significant buy-in from colleagues	−0.458 (0.385)	1.414	0.633
Significant interest on the part of potential participants	1.326 (0.384)**	11.919	3.766
Monetary compensation to facilitators	0.460 (0.643)	0.513	1.585
Support from leadership	0.051 (0.381)	0.018	1.052
Monetary compensation to participating individuals	0.663 (0.895)	0.548	1.940
Helped you identify co-facilitators	−0.374 (0.411)	0.827	0.688
Other support	0.485 (0.681)	0.509	1.625
**Institutional Inner Setting Barrier Variable**			
Lack of recognition of my implementation efforts during merit-raise processes	0.249 (0.751)	0.110	1.283
Lack of recognition of my implementation efforts during promotion processes	−0.063 (0.770)	0.007	0.939
Lack of recognition of my implementation efforts during annual review processes	−0.606 (0.749)	0.656	0.545
Lack of protected time (within my existing role) for implementation	−0.060 (0.424)	0.020	0.941
Lack of protected time (within their existing roles) for participating individuals	−0.253 (0.418)	0.365	0.777
Lack of administrative support for training (e.g., materials, food, space)	−0.993 (0.588)	2.855	0.370
Lack of administrative support for recruitment	−0.158 (0.705)	0.050	0.854
No publicly communicated support for implementation	0.508 (0.582)	0.760	1.661
No assistance identifying training participants	0.472 (0.696)	0.461	1.603
Lack of buy-in from colleagues	−0.332 (0.521)	0.406	0.717
Lack of interest on the part of potential participants	0.626 (0.479)	1.706	1.869
Lack of monetary compensation to facilitators	−0.326 (0.534)	0.373	0.722
Lack of support from leadership	0.034 (0.552)	0.004	1.035
Other competing trainings offered on campus	0.524 (0.488)	1.154	1.690
Lack of monetary compensation to participating individuals	−0.015 (0.542)	0.001	0.986
No assistance identifying co-facilitators	−0.311 (0.795)	0.153	0.733
Other barriers	−1.916 (0.712)**	7.254	0.147
Constant	1.194 (0.516)	5.349	3.299

B is the estimated coefficient, with standard error (SE). If the Wald statistic is significant, then the parameter is useful to the model. Exp(B) is the predicted change in odds for a unit increase in the predictor. When Exp(B) is less than 1, increasing values of the variable correspond to decreasing odds of the event’s occurrence. When Exp(B) is greater than 1, increasing values of the variable correspond to increasing odds of the event’s occurrence. The probability of the dependent response between implementer group and non-implementer group was predicted using B(SE), Wald, and Exp(B) with *p < 0.05, **0.001<p < 0.01, ***p < 0.00.

Institutional inner setting support and institutional barrier items from the survey were also examined across implementation status. Among the *institutional support factors*, two items were found to significantly influence implementation status: (1) administrative support for training and (2) significant interest on the part of potential participants (Table 6). Respondents who reported receiving those supports were more likely to report implementing research mentor training. Among the *institutional barrier factors*, one item was found to be significant: Other barriers, where respondents had the option to write in an additional barrier that was not listed in the survey (Table 6). Respondents who reported Other barriers were less likely to report implementing research mentor training. Examples of other barriers noted include lack of centralized coordination, misalignment with the unit/department mission, and an emphasis on lack of time for implementation.

## Discussion

Until now, research on the effectiveness of the *Facilitating Entering Mentoring* dissemination model had been focused on the innovation itself, the individuals, and the implementation process [11,13]. Because the inner setting is the environment in which the innovation is implemented, we felt that it was crucial to use the CFIR model to examine factors across domains, especially at the institutional inner setting level, which may support or impede implementation efforts. While the *Facilitating Entering Mentoring* workshop is designed to equip participants with robust strategies for implementation, organizations vary in structural characteristics and culture, which impact the delivery of the innovation. Examining the support factors and the barrier factors separately provided an opportunity to thoroughly explore differences between the implementer and non-implementer groups. Our findings support this, with results indicating significant differences in institution-level factors but no significant differences in individual-level factors (confidence in facilitating, interest in facilitating, etc.) between the implementer and non-implementer groups. This lack of significant differences across individual implementation factors was not surprising. The *Facilitating Entering Mentoring* workshop has been proven to build implementation confidence to provide participants with access to a robust set of materials and resources and results in effective implementations [11–13]. Further, the high demand for mentorship education and the increasing number of available participants expressing interest in attending contribute to this finding.

Several differences were found across individual demographic categories, however. Specifically, individuals who reported their title as a postdoctoral fellow and non-tenure track instructor were less likely to report implementation. Given that institutional roles are interconnected with the power granted to individuals in those roles within a research organization, these findings are not surprising. Among the demographic categories, race and gender were not statistically significant factors in our analysis. We hope to examine these data in future studies that incorporate a larger sample size and apply an intersectional lens to explore power dynamics beyond individual identities.

Between the two groups, implementers were significantly more likely than non-implementers to identify inner setting factors as supportive. These included factors in the CFIR structural characteristic construct (protected time, monetary support to facilitators and participants), the tension-for-change construct (buy-in from colleagues, interest from participants, support from leadership), and resources construct (administrative support). Implementers were also more likely to identify “other supports” as helpful to their implementation efforts, such as support from other departments on campus, external grant funding, campus initiatives promoting mentorship, and involvement from administrative leaders. Support from leadership and stakeholders has been identified in other studies as a key facilitator to implementation initiatives and is aligned with other research suggesting that perceived implementation leadership and implementation climate are critical for successful implementation of evidence-based interventions [37–39].

The non-implementer group was significantly more likely to report that their institution would provide recognition of implementation efforts during promotion processes. Given our finding that instructors and postdocs were less likely to report implementation, and individuals in those roles are at earlier career stages, it makes sense that a perceived benefit of receiving recognition of work in promotion processes would be more salient. Perhaps as predicted, the non-implementer group was more likely to report experiencing barriers during the implementation planning process.

The list of reported perceived institutional barriers is numerous, with the most significant barriers including a lack of protected time for implementation, lack of administrative support for training logistics and recruitment, lack of monetary compensation, no assistance identifying co-facilitators, and other barriers such as lack of centralized coordination, misalignment with unit/department mission, and an emphasis on competing demands on time. These barriers are part of the inner setting, suggesting that institutional and department factors are key contributors (or deterrents) to implementation. These findings provide an opportunity to consider how institutional leaders can support research mentor training facilitators at all levels, including how to address positionality challenges. These high- and mid-level leaders play an important role in determining how the work of advancing the culture of mentorship is valued and recognized in the inner setting, which may be especially key for early career stage implementers, such as the postdoctoral fellows and non-tenure track instructors who were found to be less likely to implement in this study.

The institutional barriers that were *not found significant* by the non-implementer group include lack of recognition of implementation efforts during merit-raise processes, lack of buy-in from colleagues, lack of interest on the part of potential participants, and lack of support from leadership. Perhaps not surprisingly, the implementer group reported no institutional barrier factors impeding implementation.

While data from this study were acquired from participants who completed the *Facilitating Entering Mentoring* workshop before the COVID-19 pandemic, many of the institutional barriers such as lack of protected time and administrative would likely still be applicable, despite *Entering Mentoring* increasing in accessibility due to online delivery methods. Since the pandemic, *Facilitating Entering Mentoring* workshops are hosted by CIMER in both online and in-person formats. Although implementation surveys have not been administered to the online cohorts, satisfaction and learning outcome data suggest similar results. It is possible that access to tools and resources to implement online may increase implementation rates, which could be strengthened by more empirical research.

The results of this study provide a window into the individual and institutional factors that promote and limit the implementation of *Entering Mentoring*-based mentor training. Overall, these data emphasize the importance of inner setting variables and the role of institutional support in promoting implementation efforts. Findings are consistent with other research on barriers to facilitation across a variety of education and healthcare settings and highlight the importance of organizational support and engagement of stakeholders [38,40–42]. Data overall support the effectiveness of the *Facilitating Entering Mentoring* workshop as a mechanism to disseminate evidence-based mentor training.

The authors hope this work will contribute to efforts to scale evidence-based research mentorship interventions in research-based institutions. A critical connection has been noted between researchers’ ability to engage in productive scientific collaboration and the importance of structured mentoring experiences [43–46]. More widespread use of *Entering Mentoring* has the promise to support the research capacity development in university, national laboratory, governmental, and industrial contexts.

## Conclusion

The CFIR model provides a useful framework for understanding factors that promote and limit implementation outcomes of an evidence-based research mentor training intervention. Factors associated with the inner setting domain were found to have the most impact on implementation efforts, with administrative support, leadership support, and interest from potential participants being the most significant predictors of implementation. Additionally, implementers were more likely to report receiving institutional support compared with those who did not implement the intervention. Non-implementers were more likely to report barriers, such as lack of protected time for implementation, lack of administrative support, and lack of monetary compensation.

Overall, the findings support the effectiveness of the *Facilitating Entering Mentoring* workshop as a strategy to support implementation efforts and as a mechanism to disseminate evidence-based mentor training on the national level and increase the diversity of the translational science workforce. Additionally, this study demonstrates the important role of the institution and institutional leadership in fostering an environment that values efforts to advance cultures of mentorship.

### Limitations

Although this study provides a useful framework for understanding factors that promote and limit implementation outcomes, several possible limitations are acknowledged:

1. Self-reported data: The data on factors promoting and limiting implementation of mentor training were self-reported by *Facilitating Entering Mentoring* participants and prone to bias, such as recall errors. It is also likely that those who implemented mentor training were more likely to complete the survey compared to the non-implementer group. However, demographic data collected from self-report survey data are representative of the demographic distribution of the *Facilitating Entering Mentoring* attendees overall.
2. Intervention Implementation Survey response rate: Although the 30.09% response rate is comparable with what is expected with web-based surveys [47], there is the possibility that the data are not representative of the entire population. However, the response rates for the annual surveys were similar, including the final survey, which was administered in June 2020 and had a slightly higher response rate than the average.
3. Financial support of participants: All participants included in this study attended a *Facilitating Entering Mentoring* workshop that was held in person and likely received financial support from their institution for travel and registration. This type of institutional support was not reflected in the survey design and could be explored in future studies, including the impact of online *Facilitating Entering Mentoring* workshops and the use of scholarships to promote accessibility.
4. Sample size: The results in this study are limited by our sample size, especially among certain demographic groups. Analyses could be strengthened by having a larger sample size to examine the data more thoroughly, including exploring differences across institution type, geographic region, department, and discipline.
5. Incomplete demographic data for gender and race/ethnicity: Some participants did not provide this information when completing the Intervention Implementation Survey. To handle the missing data for gender and race/ethnicity, dummy variables were created to indicate missingness. However, the missing data may affect the generalizability of the results, as participants who did not report these variables may differ from those who did. This could also impact our understanding of how demographic factors relate to the outcome. Future research should aim to improve data collection on these demographics and consider using more advanced methods, like multiple imputation, to address missing data.

### Future directions

Additional data gathering and in-depth analyses, such as closer examination of covariates (e.g., facilitator institution type) and experiences of facilitators who implemented during the COVID-19 public health crisis (outer setting), could be used to better understand the factors promoting and limiting mentor training implementation for both the implementer and non-implementer groups. Analyses might also be strengthened by qualitative analysis to examine open-ended survey responses. Network analysis may also be helpful to study and shape dissemination and implementation processes and outcomes. Finally, materials developed to support facilitator readiness could be examined to determine what additional approaches are needed to prepare facilitators and support their implementation efforts.
